# Violence risk and mental disorders (VIORMED-2): A prospective multicenter study in Italy

**DOI:** 10.1371/journal.pone.0214924

**Published:** 2019-04-16

**Authors:** Stefano Barlati, Alberto Stefana, Francesco Bartoli, Giorgio Bianconi, Viola Bulgari, Valentina Candini, Giuseppe Carrà, Cesare Cavalera, Massimo Clerici, Marta Cricelli, Maria Teresa Ferla, Clarissa Ferrari, Laura Iozzino, Ambra Macis, Antonio Vita, Giovanni de Girolamo, for the VIORMED-2 Group

**Affiliations:** 1 Department of Mental Health and Addiction Services, ASST Spedali Civili of Brescia, Brescia, Italy; 2 Department of Clinical and Experimental Sciences, University of Brescia, Brescia, Italy; 3 Department of Medicine and Surgery, University of Milano Bicocca, Monza, Italy; 4 Department of Mental Health, ASST Ovest Milanese, Milano, Italy; 5 Unit of Epidemiological and Evaluation Psychiatry, IRCCS Istituto Centro San Giovanni di Dio Fatebenefratelli, Brescia, Italy; 6 Department of Psychology, Catholic University of the Sacred Heart, Milano, Italy; 7 Department of Mental Health, San Gerardo Hospital of Monza, Italy; 8 Department of Mental Health, ASST-Rhodense G.Salvini of Garbagnate, Milano, Italy; 9 Unit of Statistics, IRCCS Istituto Centro San Giovanni di Dio Fatebenefratelli, Brescia, Italy; Chiba Daigaku, JAPAN

## Abstract

**Background:**

The management of mentally ill offenders in the community is one of the great challenges imposed on community psychiatry.

**Aim:**

The aim of this study was to analyze the association between sociodemographic, clinical, and psychosocial factors and violent behavior in a sample of outpatients with severe mental disorders.

**Method:**

This was a prospective cohort study with a baseline cross-sectional design used to provide a detailed analysis of patients’ profiles, followed by a longitudinal design to measure aggressive and violent behavior during a 1-year follow-up. Patients with severe mental disorders, with or without a history of violence, were enrolled in four Italian Departments of Mental Health and underwent a comprehensive multidimensional assessment.

**Results:**

The sample included 247 outpatients, for a total of 126 cases and 121 controls. Compared to controls, patients with a history of violence had a greater frequency of lifetime domestic violence, a greater lifetime propensity to misuse substances, and a higher number of compulsory admissions. The forthnightly monitoring during the 1-year follow-up did show statistically significant differences in aggressive and violent behavior rates between the two groups. Verbal aggression was significantly associated with aggression against objects and physical aggression. Moreover, outpatients with an history of violence showed statistically significant higher MOAS scores compared to both residential patients with an history of violence, assessed in the first wave of this project, and all controls.

**Conclusions:**

Patients with a history of violence had specific characteristics and showed a greater occurrence of additional community violence during a 1-year observation period. Our results may assist clinicians in implementing standardized methods of patient assessment and violence monitoring in outpatient mental health services and may prompt improved collaboration between different community services.

## Introduction

Several studies have investigated the association between severe mental disorders (SMDs) and violence, many of which focused on psychiatric patients admitted to or discharged from acute inpatient facilities, [1] Residential Facilities (RFs) [2], or Forensic Mental Hospitals (FMHs) [3]; other studies have assessed the risk of violence among outpatients in treatment at mental health services [4,5]. All these studies have identified several variables that may increase the risk of violence, including male gender, a diagnosis of schizophrenia, substance use disorders, and a lifetime history of violence [6]. However, only a few studies prospectively assessed the frequency of aggressive and violent behavior among patients in different treatment settings, such as outpatient care and RFs: the latter in many countries have replaced mental hospitals for long-term care [7,8].

In Italy, very few retrospective studies have been conducted to investigate the clinical and sociodemographic profiles of patients at risk for violence or to assess the frequency and severity of aggressive and violent behavior among psychiatric outpatients [9–11]; none of these studies that have been conducted used standardized multidimensional evaluation tools. This represents a serious problem, especially when considering the current structure and provision of mental healthcare in Italy [12,13], which includes the recent closure of all FMHs [14], with the consequent increase in the number of mentally ill offenders who are in outpatient treatment at ordinary Departments of Mental Health (DMHs).

In this study, we aimed to investigate the prevalence of aggressive and violent behavior in a large sample of outpatients with SMDs and their associated factors. First, we examined the sociodemographic, clinical, and treatment-related characteristics of psychiatric outpatients with SMDs with a history of violence (i.e., cases), compared to controls. Second, we measured aggressive or violent behavior exhibited by outpatients during a 1-year period, using a standardized tool, and analyzed a variety of risk and protective factors. An additional aim was to compare the frequency of aggressive and violent behavior (over a 1-year period) among (a) residential patients (VIORMED-1 Study) assessed in wave 1 of this project [15], (b) outpatients (VIORMED-2 Study), both with a history of violence, and (c) residential and outpatients without any history of violence.

## Method

### Design overview and participants

The Violence Risk and Mental Disorder (VIORMED-2) is a prospective cohort study, with a baseline cross-sectional comparative design, followed by a 1-year follow-up observation period.

Outpatient recruitment was carried out at four DMHs in Lombardy (Northern Italy). In each of the four DMHs, there are four community mental health centers that provide outpatient care; the participating sites have a catchment area with an average population of 351,400 (±32,366.70) (see S1 Table). The average number of outpatients receiving care is 4,206 (±360.13).

Recruitment started in the second half of 2015 and study participants were then consecutively recruited during six months. Inclusion criteria were a primary psychiatric diagnosis and age between 18 and 65 years. Exclusion criteria included a diagnosis of organic mental disorder, mental retardation, dementia, or sensory deficits.

Cases were recruited first. The selection of these patients was based solely on a comprehensive and detailed documentation (as reported in clinical records) about a history of violent behavior(s). Violent patients had to meet any of the following criteria: (i) to have been admitted at least once to a FMH for any violent acts against people and then discharged; and/or (ii) to have a documented lifetime history of violent acts against people in the last 10 years (as reported in the official clinical records), which caused physical harm to the victim, or having committed armed robbery, pyromania, or sexual violence; these behaviors led to legal prosecution or to arrest. The control group included patients who did not meet any of these three conditions during their lifetime.

All participants provided written informed consent before entering the study. Before signing consent, the treating clinician with the local research assistant provided the potential participant with detailed information about the observational nature of the study, of the study aims and methods. The participant information sheets and consent/assent forms made explicit the voluntary nature of subjects’ involvement and the possibility to withdraw from the study at any time. There were 6 patients who had a legal representative: 3 ‘cases’ and 1 control in Garbagnate; 1 control in Legnano and 1 case in Brescia. In these six cases the informed consent was initially sought from the legal representative, and then from the patient. Even if the legal representative gave consent but the patient refused, that person was not included in the study. Patients were assessed with several standardized instruments within 14 days of recruitment. The purpose of the observation follow-up period, which started once patients had completed baseline assessment, was to measure and quantify patients’ aggressive and violent behavior. Ethical approval was granted by the ethical committee of the coordinating center (IRCCS Saint John of God, *Fatebenefratelli;* n° 64/2014) and by ethical committees of all other recruiting centers.

### Measures and assessments

A specific patient schedule was developed to collect information on selected sociodemographic characteristics, clinical and treatment-related factors, and the history of violence (to be completed for cases only). The Structured Clinical Interview for DSM-IV Axis I (SCID-I) [16] and Axis II (SCID-II) [17] were administered to confirm clinical diagnoses. The global concordance was evaluated by Cohen’s K index, and it was K = 0.93 for Axis I and K = 0.65 for Axis II; the latter value (which, according to Landis and Koch, should be considered substantial) [18] is in the range of Cohen’s k value found in Italy by Maffei et al. [19] in a sample of 231 consecutively admitted in- and outpatients assessed with the SCID-II. Symptom severity and psychosocial functioning were assessed using the Brief Psychiatric Rating Scale-Expanded (BPRS-E) [20], and the Specific Levels of Functioning scale (SLOF) [21].

Aggressiveness, impulsiveness, and hostility were evaluated through a set of self-reported measures, notably (a) the Brown-Goodwin Lifetime History of Aggression (BGLHA) [22], an 11-item questionnaire assessing lifetime aggressive behavior across two stages of life (adolescence and adulthood) by directly asking how many times the aggressive behavior occurred for each item; (b) the Buss-Durkee Hostility Inventory (BDHI) [23], a 75-item questionnaire containing eight subscales (e.g, direct and indirect aggression, irritability, negativism, resentment, suspiciousness, verbal aggression and guilt) and producing an index of inhibition of aggression (a higher score indicating more hostility); and (c) the Barratt Impulsiveness Scale (BIS-11) [24], a 30-item 4-point Likert scale questionnaire that investigates personality and behavioral impulsiveness, with scores ranging from 30 to 120 (a higher score indicating more impulsiveness). The State-Trait Anger Expression Inventory 2 (STAXI-2) [25], which includes 57 items grouped into six scales (state and trait anger, anger directed inside and outside, control and expression of anger) plus an anger expression index and an overall measure of total anger expression (a higher score indicates more anger) evaluated on a 4-point Likert scale, was employed to provide specific measures of anger.

Aggressive and violent behavior exhibited by patients during the 1-year follow-up were rated every fifteen days with the Modified Overt Aggression Scale (MOAS) [26], for a total of 24 MOAS evaluations for each patient. All MOAS evaluators (treating clinicians and other mental health staff, and family relatives) were very familiar with the patients and had daily, or very frequent, contact with them. The MOAS includes four aggression subdomains: verbal, against objects, against self, and physical-interpersonal. A score from 0 to 4 is assigned: 0 indicating no aggressive behavior and higher scores showing increasing severity. The score in each category is multiplied by a factor assigned to that category, which is 1 for verbal aggression, 2 for aggression against objects, 3 for aggression against self, and 4 for aggression against other people. The total weighted score for each evaluation ranges from 0 (no aggression) to 40 (maximum grade of aggression); since there were 24 ratings during a 1-year period, the individual MOAS total score for that time period ranged from 0 to 960. We will subsequently refer to the weighted MOAS total score (our primary outcome) simply as the MOAS score.

Treatment compliance was rated with the Medication Adherence Rating Scale (MARS), a 10-item self-report questionnaire validated in patients with psychosis [27].

### Statistical analyses

To compare categorical data, a χ^2^ test or the exact Fisher’s test, whenever appropriate (n<5 in any cell), were used. For quantitative data, ANOVA or a nonparametric Mann Whitney test were used. The normality assumption was verified by visual inspection of the variable distribution through QQ-plots and box plots in addition to Shapiro-Wilk and Kolmogorov-Smirnov tests.

For the BPRS-E, an exploratory factor analysis was used to identify the main scale domains. Factor extraction was performed by varimax rotation, and the number of factors was determined through Kaiser’s criterion (i.e., eigenvalue ≥1) and through visual inspection of the screen plot. Factor loadings with the highest value (among extracted factors) were considered to contribute sufficiently to the overall variability accounted for in each factor.

Monitoring of aggressive and violent behavior was carried out by analyzing MOAS scores across all 24 evaluations, and their trends were estimated with the smoothing-splines method [28]. Given the non-Gaussian distribution of the MOAS scores (skewed and zero-inflated distribution), generalized estimating equation models (GEE) with tweedie distribution and log-link function were used to analyze MOAS repeated measurements.

Finally, the analyses of predictive factors for violence were performed by adopting generalized linear models (GLMs) with tweedie distribution and log-link function (MOAS score—total and subscales—used as the dependent variable and all other measurements as independent ones). Model goodness of fit was evaluated by Akaike information criterion (AIC: the lower value indicates a better model). The beta coefficients are reported as exponential reparameterization of the standardized ones for easier interpretation.

All tests were two-tailed with statistical significance set at p = 0.05. All data were coded and analyzed using the Statistical Package for Social Science (SPSS, version 21 for Windows Chicago, Illinois 69606, USA), and R: A language and environment for statistical computing (R Core Team, 2015), R Foundation for statistical Computing, Vienna, Austria.

## Results

### Sample

To estimate the minimum sample size based on significant change (baseline-follow-up) differences in the MOAS Total Score between violent and control groups we relied on two studies (Margari et al. [26] and Mauri et al. [29]), indicating that it was necessary to enroll 232 patients (116 cases and 116 controls). However, our main aim was to recruit a larger sample to prevent possible dropouts, so that at the end of the recruitment period (6 months) we enrolled 15 more patients (10 cases and 5 controls). Among the 274 patients who were asked to join the study, 27 (9.8%) refused; therefore, the total study sample included 247 outpatients with a primary diagnosis of SMDs: 126 of them had a lifetime history of violence (i.e., cases) and 121 had no such history (i.e., controls). The two groups did not differ in age, gender, nationality, marital status, or occupation. Compared to the controls, the cases had a lower educational level (X^2^ = 4.3, p = 0.038), spent more time doing nothing (more than 3 hours per day; X^2^ = 7.9, p = 0.005), and had received less social support during the past year (X^2^ = 4.0, p = 0.046). Regarding a lifetime history of violence, the proportion of participants who had witnessed or were involved in at least one episode of domestic violence was higher among cases (X^2^ = 20.2, p < 0.001). The sociodemographic information is presented in Table 1.

**Table 1:** Socio-demographic characteristics of patients with an history of violence and controls.

	Violent group		Controls		Test*	*p-value*
	N = 126	%	N = 121	%		
**Gender**						
Male	103	81.7	90	74.4	1.96	0.161
Female	23	18.3	31	25.6		
**Nationality**						
Italian	121	96.0	119	98.3	1.20	0.240
Others	5	4.0	2	1.7		
**Age**						
18–35	20	15.9	25	20.8	2.80	0.247
36–50	70	55.6	54	45.0		
51+	36	28.6	41	34.2		
**Marital status**						
Married or cohabiting	51	40.5	47	38.8	0.07	0.793
Single	75	59.5	74	61.2		
**Education**						
Low level	82	65.1	63	52.1	4.31	0.038
Medium-high level	44	34.9	58	47.9		
**Occupation**						
Employed	52	41.6	60	50.4	1.91	0.167
Unemployed	73	58.4	59	49.6		
**Economic independence**						
Yes	54	44.3	55	47.0	0.18	0.670
No	68	55.7	62	53.0		
**Social support in the last year**						
Present	86	72.3	94	83.2	3.97	0.046
Not present	33	27.7	19	16.8		
**Time spent doing nothing**						
Less than 3 h per day	46	37.4	66	55.5	7.94	0.005
More than 3 h per day	77	62.6	53	44.5		
**Episodes of violence in family**						
Yes	40	34.2	11	9.6	20.21	<0.001
No	77	65.8	103	90.4		

* Chi-squared test or Exact Fisher’s test (when n <5 in at least one cell).

The most frequent primary diagnoses included schizophrenia spectrum disorders (up to 41.3%) and personality disorders (up to 28.1%). The mean duration of illness was 17.7 years (SD = 10.5) for the violent group and 16.0 years (SD = 10.0) for the control group (F = 1.8, p = 0.186). The mean age of patients at their first contact with mental health services was 28.6 years (SD = 10.4) for the violent group and 29.8 years (SD = 11.5) for the control group (F = 0.7, p = 0.396). Cases had a higher number of past compulsory admissions to psychiatric hospital wards (X^2^ = 19.8, p < 0.001) and were less able to collaborate with treating clinicians during the previous year (X^2^ = 5.1, p = 0.023). With regard to lifetime substance use disorders, higher comorbid rates were found among cases compared to controls (X^2^ = 8.3, p = 0.004), while there were no between-group differences in alcohol misuse (X^2^ = 2.1, p = 0.145). The groups did not differ even in recent substance use disorders over the previous 12 months as reported by treating clinicians. No statistical differences were observed regarding medication (present/absent) or the presence/absence of symptoms during the previous 2 years. Clinical and treatment-related characteristics are reported in Table 2.

**Table 2:** Baseline clinical characteristics of patients with an history of violence and controls.

	Violent group (N = 126)			Controls (N = 121)			Test*	*p-value*	
	Mean	SD		Mean		SD			
**Illness duration (Years)**	17.73	10.52		15.99		9.98	1.76	0.186	
**Age of first contact with DMHs (Years)**	28.63	10.36		29.81		11.48	0.72	0.396	
	N		%	N	%		Test*		*p-value*
**Primary diagnosis by the treating clinician**									
Schizophrenia	52	41.3		52		43.0	34.20	0.331	
Personality disorder	47	37.3		34		28.1			
Bipolar disorder	13	10.3		14		11.6			
Anxiety/Mood disorders	14	11.1		21		17.4			
**Current comorbidity with substance use disorders**									
Alcohol	9	7.3		4		3.3	2.44	0.295	
Other substances	13	10.5		17		14.0			
None	102	82.3		100		82.6			
**Lifetime alcohol use disorders**									
Yes	39	31.0		27		22.7	2.12	0.145	
No or occasional	87	69.0		92		77.3			
**Lifetime substance use disorders**									
At least one	47	37.9		25		21.0	8.31	0.004	
None	77	62.1		94		79.0			
**Alcohol use disorder in the last 12 months**									
Yes	38	30.2		30		24.8	0.89	0.345	
No	88	69.8		91		75.2			
**Misuse of at least one substance in the last 12 months**									
Yes	19	15.2		12		9.9	1.56	0.212	
No	106	84.8		109		90.1			
**Lifetime compulsory admissions**									
None	66	54.1		88		72.7	19.81	<0.001	
1–3	40	32.8		33		27.3			
≥4	16	13.1		0		0.0			
**Access rate to outpatient community services**									
1 time per month	89	70.6		72		59.5	3.37	0.066	
Less than 1 time per month	37	29.4		49		40.5			
**Capability of collaboration in the last year**									
Collaborating	106	89.1		115		96.6	5.13	0.023	
Non-collaborating	13	10.9		4		3.4			
**Psychopharmacological treatment**									
Yes	115	92.0		115		95.0	0.94	0.334	
No	10	8.0		6		5.0			
**Psychopathological symptoms (last 2 years)**									
Absent	78	67.8		80		67.8	0.00	0.996	
Persistent	37	32.2		38		32.2			

DMHs = Departments of Mental Health

* ANOVA for continuous variables; Chi-square test or Fisher’s exact test (n<5 in at least one cell) for categorical variables.

### History of violence

Cases committed a large number of violent offenses, including physical aggression (87.2%), stalking (3.2%), sexual violence (2.4%), armed robbery (1.6%), murder (1.6%), attempted murder (0.8%), and other violent acts (3.2%). In more than one-fourth of cases, violent behavior was committed in the presence of psychotic symptoms, and in 20.5% of the instances the offenders were under the influence of alcohol. The history of violence was more frequently due to an episode of impulsive violence (92.4%). Victims of violence were more frequently the patients’ parents or partners (respectively 28.0% and 24.6%), followed by clinical staff (6.8%), patients’ friends (6.8%), other relatives (6.8%), other patients (2.5%), or others (24.6%). The large majority of patients (88.8%) recognized their acts as violent, while the remaining 11.2% denied the violent nature of the offenses. Almost one-fourth (23.4%) of the violent patients were arrested for the violent offenses; 72.8% of patients already had a diagnosis of SMD at the time of their violent offense, and 67.5% were under care at the local DMH. Cases obtained higher scores on the BGLHA (mean score: 40.4, SD = 12.4 for cases vs. 33.6, SD = 9.7 for controls; p < 0.001) (Table 3).

**Table 3:** Baseline assessment: clinician-administered assessment tools and self-reports.

	Violent group (N = 126)		Controls (N = 121)		Test*	*p-value*
	Mean	SD	Mean	SD		
**BPRS-E**						
Affect-Anxiet*y*	11.17	4.11	10.70	4.19	–0.73	0.463
Activation	11.67	4.75	9.55	3.09	–3.81	<0.001
Negative Symptoms	5.48	3.15	5.26	2.72	–0.57	0.572
Psychotic Symptoms	12.38	5.47	10.88	3.50	–1.16	0.247
Total score	41.01	11.75	36.85	8.89	–2.43	0.015
**SLOF**						
Physical functioning	24.13	1.44	24.25	1.34	0.83	0.406
Self-care	33.24	3.14	33.37	3.12	0.44	0.663
Interpersonal relationships	23.90	5.94	24.93	5.63	1.46	0.143
Social acceptability/adjustment	23.69	4.03	26.96	2.70	6.81	<0.001
Activities	48.51	7.39	49.80	6.02	1.03	0.303
Work skills	21.51	6.57	23.16	6.11	1.91	0.056
**BGLHA**						
Total score	40.38	12.44	33.59	9.68	–3.91	<0.001
**BIS-11**						
Attentional impulsiveness	15.40	4.32	14.51	3.80	–1.47	0.142
Motor impulsiveness	22.64	4.86	21.37	4.55	–1.85	0.064
Non-planning impulsiveness	27.21	5.38	26.42	5.38	–1.21	0.228
Total score	64.81	11.56	62.10	10.41	–1.62	0.105
**BDHI**						
Assault	4.58	2.61	4.43	2.45	0.17	0.683
Indirect aggression	4.62	2.25	4.56	1.73	0.05	0.822
Irritability	4.11	2.60	4.00	2.29	0.09	0.759
Negativism	2.63	1.57	2.31	1.54	2.02	0.157
Resentment	3.90	1.98	3.89	2.35	0.00	0.976
Suspicion	4.13	2.38	4.28	2.62	0.15	0.702
Verbal aggression	6.61	2.82	6.25	2.49	0.82	0.367
Guilt	4.88	2.33	4.36	2.33	2.26	0.135
*Total score*	35.58	14.58	34.62	12.57	0.19	0.665
**STAXI-2**						
State anger	33.44	200.23	31.47	17.68	–0.29	0.770
Feeling angry	24.48	23.79	25.67	23.39	–0.10	0.919
Feel like expressing anger verbally	24.58	24.46	24.42	21.88	–0.23	0.818
Feel like expressing anger physically	23.32	23.44	23.73	22.26	–0.04	0.969
Trait anger	32.97	19.42	29.07	16.02	–1.29	0.197
Angry temperament	25.42	23.82	23.87	21.12	–1.43	0.153
Angry reaction	25.38	23.34	23.14	19.14	–0.72	0.474
Anger expression-out	30.14	20.99	26.17	16.41	–1.74	0.083
Anger expression-in	30.93	19.96	31.77	19.14	0.15	0.878
Anger control-out	27.91	13.61	33.00	15.37	2.72	0.006
Anger control-in	31.20	15.47	35.05	16.63	2.06	0.040
Anger expression index	46.45	16.83	39.91	15.15	–2.84	0.005

BDHI = Buss-Durkee Hostility Inventory; BGLHA = Brown-Goodwin Lifetime History of Aggression; BIS-11 = Barratt Impulsiveness Scale; BPRS-E = Brief Psychiatric Rating Scale; SLOF = Specific Levels Of Functioning; STAXI-2 = Scale State-Trait Anger Expression Inventory 2.

* Mann-Whitney test for BPRS-E, SLOF, BGLHA, BIS, STAXI-2; ANOVA for BDHI.

### Psychopathology

The exploratory factor analysis of the BPRS-E suggested a four-factor structure named affect-anxiety, activation, negative symptoms and psychotic symptoms (S2 Table). This BPRS-E structure was similar to the previously published [30]. Cases showed higher symptom severity than controls (Table 3). We found a statistically significant difference in the BPRS-E total score between the two groups (mean score: 41.0, SD = 11.7 for cases vs. 36.9, SD = 8.9 for controls; p = 0.015) and different scores in the BPRS-E activation subdomain (mean score: 11.7, SD = 4.8 for cases vs. 9.6, SD = 3.1 for controls; p < 0.001).

### Psychosocial functioning and treatment compliance

Although cases had lower scores on all SLOF domains, a statistically significant difference was found for the social acceptability subscale (mean score: 23.7, SD = 4.0 for the violent group vs. 27.0, SD = 2.7 for controls; p < 0.001) (Table 3).

With regard to treatment compliance, patients with an history of violence had a significantly lower MARS average score (6.87, SD = 2.31) compared to controls (7.51, SD = 1.73) (t-test = -2.06, p = 0.041).

### Impulsiveness and anger

We did not find any differences between groups in the BDHI and BIS-11 scores. A statistically significant difference was found on two STAXI-2 subscales and for the Anger Expression Index: (i) anger control-out (mean score: 27.9, SD = 13.6 for the violent group vs. 33.0, SD = 15.4 for the control group; p = 0.006); (ii) anger control-in (mean score: 31.2, SD = 15.5 for the violent group vs. 35.1, SD = 16.6 for the control group; p = 0.040); (iii) Anger Expression Index (mean score: 46.5, SD = 16.8 for the violent group vs. 39.9, SD = 15.2 for the control group; p = 0.005). The mean total scores (including subscale scores) for the BDHI, BIS-11, and STAXI-2 in both groups are shown in Table 3.

### Aggressive and violent behavior during the 1-Year follow-up

**Fig 1:**
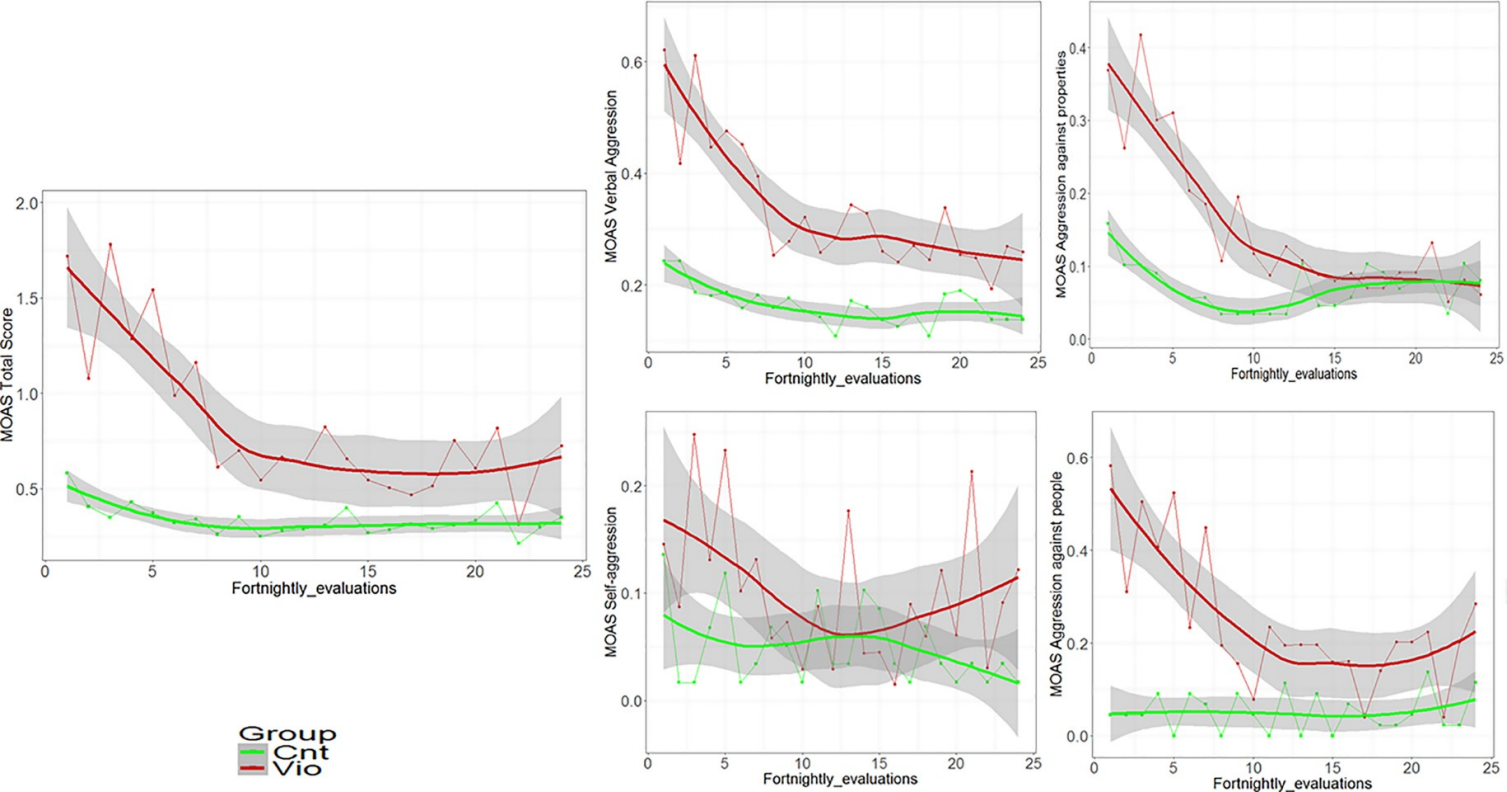
Longitudinal evaluation of MOAS Total and subscales scores during follow-up in cases and controls. Trend estimated through Smoothing Spline functions with corresponding 95% confidence bands.

Fifteen patients (11 cases and 4 controls) had more than two missing MOAS and so were not considered in these analyses. Patients with up to two missing MOAS evaluations were computed by the moving average estimation method. Compared to controls, cases displayed statistically greater scores on the MOAS total score (mean = 25.7, SD = 36.3 for the violent group and mean = 8.4, SD = 17.4 for controls; *U =* -4.7, p < 0.001). The MOAS subratings were also higher for the violent group when compared to controls. This was true for MOAS verbal aggression (mean = 10.2, SD = 12.1 vs. mean = 4.8, SD = 8.5; U = -4.1, p < 0.001), MOAS aggression against objects (mean = 4.7, SD = 8.4 vs. mean = 1.7, SD = 5.6; U = -3.9, p < 0.001), MOAS physical aggression (mean = 7.4, SD = 17.0 vs. mean = 1.0, SD = 5.0; U = -5.1, p < 0.001), and MOAS self-aggression (mean = 3.3, SD = 10.8 vs. mean = 0.8, SD = 3.9; U = -1.8, p = 0.067). Since the specific interest of this study was aggressive and violent behavior, we focused only on the first three subscales. Cases showed a quickly decreasing trend on MOAS scores, while controls had an almost stable trend (see Fig 1). Moreover, trends were statistically different between cases and controls for MOAS total scores. For the verbal and physical aggression scores and MOAS aggression against objects, cases showed a different trend (confidence bands do not overlap) only up to the thirteenth observation.

A longitudinal evaluation of the MOAS subscales was performed through generalized estimating equation (GEE) with an interaction effect between time (from 1 to 24 evaluations) and group, and showed significantly different trajectories for aggression against objects (p = 0.002) and physical aggression (p = 0.006), but not for verbal aggression (p = 0.191) (see S3 Table).

Further analyses for gender-specific differences in the frequency of aggressive and violent behavior, measured by the total MOAS, showed no significant differences between males and females (see S1 Fig).

### Predictors of aggressive and violent behavior

With regard to the relationship between the three subscales, verbal aggression was a significant predictor of aggression against objects (p < 0.001) and of interpersonal violence (p < 0.001), while aggression against objects was a significant predictor (p < 0.001) of interpersonal violence.

Generalized Linear Models with MOAS total score as a dependent variable and clinical and socio-demographic features, group and their interaction as independent variables were applied to detect which MOAS predictors had different effect in the two study groups. In order to quantify these differentiated (between the two groups) effects (evaluated by beta coefficients) we computed GLMs for the two groups separately.Results showed that the negativism score of the BDHI was the best (in terms of AIC) predictor of violent behavior (p = 0.039, AIC = 1195.3). A lower MOAS total score was predicted by higher levels of negativism (which in this instrument evaluates oppositive and conflictual behaviors) among cases (β_cases_ = 0.81).

Other predictors of the MOAS total score included the SLOF social acceptability score (p < 0.001, AIC = 1521.0), with higher total MOAS scores predicted by lower levels of social acceptability among both cases and controls (but with different strength between groups: β_cases_ = 0.92, β_controls_ = 0.75). Both MOAS verbal aggression and MOAS aggression against objects were inversely associated with the SLOF social acceptability score (respectively, p = 0.002, AIC = 1272.0, β_cases_ = 0.91, β_controls_ = 0.77; and p = 0.012, AIC = 700.5, β_cases_ = 0.89, β_controls_ = 0.71). MOAS physical aggression was predicted by BDHI total score (p = 0.036, AIC = 325.7), with higher strength in controls with respect to cases (β_cases_ = 0.98, β_controls_ = 1.11). An inverse association was found between MOAS physical aggression and SLOF social acceptability (p = 0.012, AIC = 556.5), in controls only (β_controls_ = 0.69). Table 4 shows all risk and protective factors for violent behavior (recorded during the follow-up period) with a significant different effect between the two study groups.

**Table 4:** Predictors of aggressive and violent behavior: generalized linear models (GLMs) with interaction effect between variables and groups (all sample), and corresponding GLMs for the two groups.

	All sample		Case patients group	Control group
	*p-value*	AIC	β_cases_	β_controls_
**Total MOAS**				
BDHI Negativism	0.039	1195.3	0.81**	1.08
SLOF Social acceptability/adjustment	<0.001	1521.0	0.92**	0.75**
BPRS-E Total Score	0.019	1547.3	1.01	1.06**
Lifetime misuse of substances *(No vs Yes)*	<0.001	1554.5	0.91	0.18**
Time spent doing nothing	0.008	1570.1	0.51**	1.43
BPRS-E Activation	0.001	1575.5	1.05**	1.23**
Age	0.014	1576.7	0.98**	0.94**
Primary diagnosis	0.023	1578.4	#	#
Familiarity with psychiatric illness *(Yes vs No)*	0.063	1583.8	1.60**	0.81
Comorbidity with alcohol/substances misuse	0.028	1588.9	#	#
Illness duration	0.005	1592.3	1.00	0.95**
Misuse of alcohol in the last 12 months *(No vs Yes)*	0.003	1593.3	1.04	0.33**
**MOAS verbal aggression**				
SLOF Social acceptability/adjustment	0.002	1272.0	0.91**	0.77**
BPRS-E Total Score	0.018	1295.6	1.01	1.06**
Lifetime misuse of substances *(No vs Yes)*	<0.001	1307.1	0.89	0.25**
BPRS-E Activation	0.008	1321.5	1.06**	1.19**
Age	0.011	1329.5	0.99	0.95**
Misuse of alcohol in the last 12 months *(No vs Yes)*	0.003	1332.9	0.95	0.31**
Comorbidity with alcohol/substances misuse	0.038	1334.3	$	$
BPRS-E Psychotic Symptoms	0.046	1334.8	1.00	1.09**
Illness duration	0.023	1339.1	1.00	0.96**
**MOAS aggression against objects**				
SLOF Social acceptability/adjustment	0.012	700.5	0.89**	0.71**
Lifetime misuse of substances *(No vs Yes)*	0.001	707.4	1.40	0.16**
Illness duration	0.047	720.3	0.99	0.92**
Time spent doing nothing	0.023	721.1	0.59	3.00*
Misuse of alcohol in the last 12 months *(No vs Yes)*	0.056	722.8	0.90	0.24**
**MOAS self-aggression**				
BDHI Indirect Aggression	0.025	246,5	0.98	1.99**
STAXI Feel like expressing anger physically	0.036	339,7	1.03**	0.97
STAXI Feeling angry	0.035	340,3	1.03**	0.97
STAXI Feel like expressing anger verbally	0.048	340,6	1.03**	0.97
STAXI Anger control-in	0.045	340,9	1.04**	0.97
Familiarity with psychiatric illness *(Yes vs No)*	0.005	360,3	4.67**	0.12**
Occupation *(Yes vs No)*	0.014	361,3	5.96**	0.34
**MOAS physical aggression**				
BDHI Total Score	0.036	325.7	0.98	1.11*
STAXI Anger Expression Index	0.046	510.0	1.00	1.06**
Lifetime misuse of substances *(No vs Yes)*	0.001	549.1	0.91	0.03**
Age of first contact with DMHs	0.001	553.3	0.97	0.71**
SLOF Social acceptability/adjustment	0.012	556.5	0.99	0.69**
Misuse of alcohol in the last 12 months *(No vs Yes)*	0.039	563.8	0.53	0.06**
BPRS-E Activation	0.017	566.2	1.05	1.40**
Primary diagnosis	0.024	568.0	£	£
Time spent doing nothing	0.051	568.2	0.31**	2.02
Illness duration	0.029	570.3	1.03	0.92*

BDHI = Buss-Durkee Hostility Inventory; BPRS-E = Brief Psychiatric Rating Scale; MOAS = Modified Overt Aggression Scale; SLOF = Specific Levels of Functioning; STAXI-2 = Scale State-Trait Anger Expression Inventory 2.

*p value*: significance of the interaction term; AIC: *Akaike Information Criterion* of the GLM; βcases βcontrols: estimates of the variable effect in the two groups separately.

* Tendency towards significance (*p≤*0.1) of the beta coefficient

** Significance (*p*<0.05) of the β coefficient.

# Among cases, patients with a personality disorder are more violent than other diagnostic groups (p<0.048); among controls, patients with personality disorders are more violent than patients with schizophrenia (p<0.001). Among cases, there are no significant differences between patients with and without comorbidity of alcohol/substance abuse. Among controls, patients with a comorbidity of alcohol/substance abuse are more violent than patients who do not use alcohol and substances.

$ Among cases, there are no significant differences in verbal aggression between patients with and without comorbidity of alcohol/substance abuse. Among controls, patients with a comorbidity of alcohol/substance abuse are more verbally violent than patients who do not use alcohol and substances.

£ Among cases, there are no significant differences among different diagnostic groups; among controls, patients with personality disorders are more violent than patients with schizophrenia (p<0.047).

Additionally, univariate GLMs (without considering the group distinction between cases and controls) were performed to analyze factors associated with the MOAS scores. The best predictor of new aggressive and violent behavior(s) was the BDHI suspicion score (p = 0.030, AIC = 1156.1, β = 1.14), followed by the BGLHA total score (p = 0.002, AIC = 1208.9, β = 1.05).

Furthermore, we found different factors associated with the MOAS domains. MOAS verbal aggression was predicted by the BDHI suspicion score (p = 0.038, AIC = 991.5, β = 1.11), whereas MOAS aggression against objects was predicted by the STAXI Anger Expression Index and STAXI trait anger (respectively, p = 0.014, AIC = 665.9, β = 1.03; and p = 0.038, AIC = 676.0, β = 1.02). Only the BGLHA total score predicted MOAS physical aggression (p = 0.018, AIC = 443.7, β = 1.10). The full list of the factors associated with the MOAS scores is shown in S4 Table.

### Violence and hospital admission

During the 1-year follow-up, 23 patients (16 with an history of violence, 7 controls) were hospitalized in general hospital psychiatric wards. We compared the last MOAS score prior to hospitalization with the previous four scores (two months) and with the MOAS score immediately after discharge. As shown in Supporting Information S2 Fig, there were no statistically significant differences between the MOAS scores at these different timepoints, suggesting that in our cases, hospital admission was not due to an increase in aggressive and violent behavior (S2 Fig).

### Patients with an history of violence in different treatment settings

In the first wave of this project [15], we evaluated 139 patients living in RFs, 82 violent and 57 control subjects. We did not find any statistically significant differences in aggressive behavior rates between the two groups during the 1-year follow-up. Our conclusion was that patients with a history of violence in RFs, where treatment and clinical supervision are available, do not show higher rates of aggression and violence compared to patients with no lifetime history of violence.

**Fig 2:**
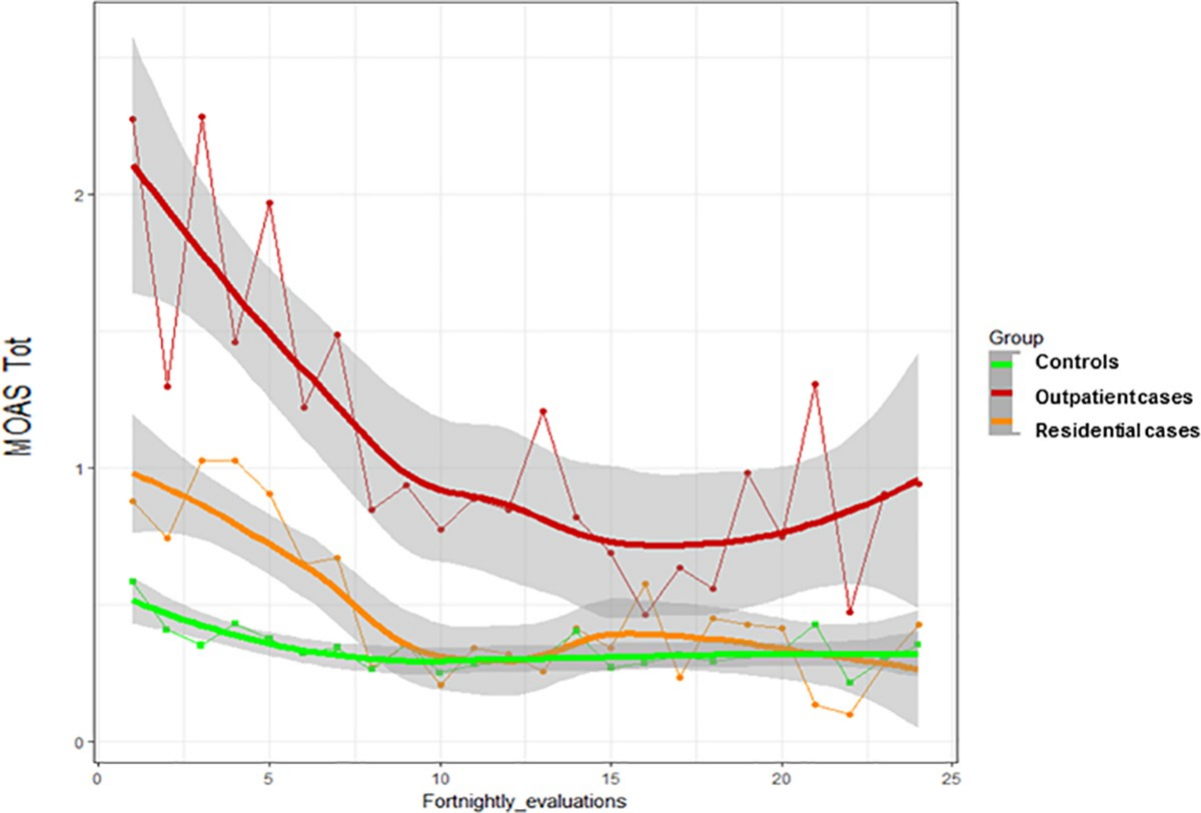
Longitudinal evaluation of MOAS Total score during the 1-year follow-up in three different clinical groups. Trend estimated through Smoothing Spline functions with corresponding 95% confidence bands.

Therefore, we decided to compare the two samples (residential and outpatients) from waves 1 and 2 to establish whether staying in a RF for patients with a history of violence has a protective effect from the risk of violent behavior. To do this, we merged the two control samples (patients with and without a history of violence), while we kept the two case samples (outpatients and residential patients) with a history of violence separated. The results of this analysis are shown in Fig 2.

Compared to both controls and residential cases, outpatient cases displayed statistically greater scores on the MOAS total score when compared to both controls and residential cases (mean = 25.7 SD = 36.3 for outpatient cases, mean = 11.4 SD = 18.0 for residential cases and mean = 8.1 SD = 17.1 for all controls; K = 32.7, p<0.001).

## Discussion

A recent Italian law (81/2014) enacted a significant reorganization of the forensic system, with the closure of the six FMHs, the opening of new small-scale high-security units, and a consequent transfer of many patients with SMD who had offended, or are at risk of offending, to ordinary DMHs (including RFs managed by these services). This change has prompted a deeper investigation into the risk of aggressive and violent behavior among patients in treatment at DMHs. To our knowledge, this is the first Italian study, and one of very few internationally, to use a large set of standardized multidimensional evaluation tools and to prospectively examine the frequency and severity of aggressive and violent behavior in outpatients with SMDs.

Previous studies examining the link between SMDs and violence have produced mixed findings [1,31]. Several factors have been suggested to explain this heterogeneity, such as different study designs, assessment tools, type of outcome(s), settings of care, and the specific national mental health policies and programmes, making it difficult to make comparisons and interpret results [6,32–34].

Our study demonstrates that outpatients with SMDs who have an history of serious violence are more likely to show higher levels (in terms of frequency and severity) of aggressive and violent behavior as compared to patients who do not have such a history, and this raises important clinical problems in terms of prevention and management.

### Are outpatients with a history of violence more likely to commit violent acts?

Our findings show that outpatients with a lifetime history of violence under care in Italian DMHs had more frequent and more severe episodes of aggression and violence compared to controls. This is in line with Italian retrospective studies on outpatients conducted after the reform of Law 81/2014 [9,10]. On the other hand, this finding is at odds with the results of the first wave of the VIORMED project, in which 82 patients living in RFs with an history of violence were compared to 57 patients with no such history. In these settings, with 24-hour cover, the difference in the frequency of aggressive and violent behavior between patients with and without a history of violence was negligibile [15]. Living in a controlled environment, with compliance granted and no possibility of substance use disorders, may have a preventive effect on aggressive and violent behavior, while life in the community, where treatment compliance is not warranted and there is a greater risk of substance use disorders, has a potential detrimental effect on the risk of recurrence. Indeed, this finding was confirmed when comparing the MOAS total scores among outpatient cases, residential cases, and all controls: while residential cases, living in a controlled environment, did not show higher scores compared to all controls, from both settings, outpatient cases displayed the highest rates of aggressive and violent behavior. To our knowledge, this is the first time ever that a study with the same prospective design compared patients with an history of violence treated in different setting, and shows a marked difference in behavioral patterns associated with different regimes of care (with higher or lower protection).

### What predicts violence?

We identified several predictive and protective factors for community violence. Social acceptability was a predictor of nonaggressive behavior, indicating that better social acceptability is associated with lower MOAS scores among both cases and controls. With specific regard to physically aggressive behavior, higher levels of anger expression did predict aggressive behavior, while hostility was predictive only among controls. Other predictors of aggressive and violent behavior that we found in our study (i.e., lifetime substance use disorders, early age at the first contact with DMHs, longer illness duration) are in line with findings from previous studies on Italian cohorts [9].

Finally, patients who showed higher levels of verbal aggression were more likely to commit physical aggression against objects or against other people, and patients who showed higher levels of aggression against objects were more likely to commit aggression against other people. This sequence seems to compose a ‘continuum’ in the occurrence of aggressive and violent behavior and offers important elements for prevention: if a patient shows increasing levels of verbal aggression, or of aggression against objects, this may indicate the need for appropriate interventions to prevent an escalation to more severe forms of violence against other people.

### Clinical differences between patients with or without violence history

Unlike previous studies on outpatient cohorts [31], we did not find any differences in many functional areas between outpatients with or without a lifetime history of physical interpersonal violence. Furthermore, at variance with some studies [35], but not other studies [9] done in outpatient samples, our cases had more severe psychopathological symptoms, as rated with the BPRS-E, compared to controls. Possible explanations for these divergent results may be related to the use of different assessment tools and to the different time elapsed from the index violent behavior. On the other hand, there is consensus in the literature concerning the association between a lifetime history of violence, a history of domestic violence, a lifetime use of substances, and a history of compulsory admissions [6].

### Impulsiveness and anger

With regard to impulsiveness, unlike previous findings [36,37], we found no differences between the two groups when rated by the BIS. Although there were differences in BGHA scores, the BDHI scores among cases were similar to those found in a study conducted among male prisoners [38]. Findings on angry feelings showed that compared to controls, cases had lower anger-control, with less ability to calm or cool down. This is in line with previous findings showing a negative association between the STAXI-2 anger control subscale and aggression [39].

### How to manage violent patients in the community

This study provides useful indications for planners and clinicians who have the relevant task of planning and managing services which currently have also to care for mentally ill offenders in Italy. While patients with a history of interpersonal violence are effectively managed in RFs [15], where treatment and clinical supervision are granted, our study shows that outpatients living in the community still pose a higher risk of reiteration of aggressive and violent behavior as compared to patients with no history of violence. It is doubtful whether current mental health services in Italy are well equipped to cope with these relevant clinical needs: despite the approval of the new law reorganizing the care for mentally ill offenders, no plans for specific training programmes of mental health workers have been developed, and residency programmes for psychiatrists in training are equally insufficient to meet these training needs; as a consequence many services are inadequately equipped to well manage difficult clinical situations raised by patients with serious histories of violence, as recognized by several authors; the relationships with judicial institutions and forensic services are equally problematic [40–42].

At the same time an active collaboration between mental health services and addiction services (which is of paramount relevance given the importance of substance use disorders as a primary risk factor for aggressive and violent behavior) is often missing, and new strategies of collaborative work involving different treatment agencies have to be developed. It will be necessary to set up appropriate monitoring systems to well understand the main unmet needs of this difficult-to-treat clinical population and identify the clinical skills which mental health workers have to learn to well manage these patients.

### Acute hospitalizations and violent behavior

During the 1-year follow-up, 23 patients (16 with an history of violence, 7 controls) were hospitalized in general hospital psychiatric wards: based on MOAS scores in the periods prior to hospitalization and after discharge, we did not find any association between hospital admission and reports of increased violent behavior. In a large survey of almost 3,000 acute patients admitted to Italian general hospital psychiatric wards, violence against people was among factors contributing to admission for a substantial proportion of hospitalized patients: this occurred in 19.3% of patients with a diagnosis of schizophrenia, 13.5% of patients with bipolar disorders, and 19.3 for patients with personality disorders or substance use disorders [43]. Given the small number of patients acutely admitted in our study, it is difficult to generalize our results, and specific investigations about the role of aggressive and violent behavior in triggering hospital admissions are warranted.

### Limitations

This study has a number of limitations. First, the duration of the observation period limited to one year may have reduced the possibility to detect new aggressive and violent episodes and, hence, to identify long-term predictors of such behavior. Second, the MOAS assessment was based on that reported by the patients’ treating clinicians, case managers, or family members and not based on a direct 24-hour observation. Thus, our results might have underestimated the occurrence of aggressive and violent behavior among outpatients, in particular because MOAS was not used to evaluate each individual aggressive episode. In any event, the limited period of observation for each MOAS rating (two weeks) makes it unlikely that relevant episodes of aggression or violence remained undetected.

## Conclusions

Our data show that outpatients with a history of violence are more aggressive than patients with no lifetime violent behavior. The management of mentally ill offenders in the community is one of the great challenges imposed on community psychiatry. Indeed, more intensive care, as found in RFs, where treatment is granted and prevention of significant substance use disorders is avoided, is associated with a substantial decrease in the frequency and severity of aggressive and violent behavior even among people with a history of violence.

Violence by the mentally ill has a profound detrimental effect on public opinion, is associated with stigma and discrimination, and places a great burden on family members, who are often the victims of such violence. Risk assessment plays a key role in the prevention and/or decrease of violent behavior [44,45]. This process is preliminary and linked to an accurate diagnosis, which defines the target population [46], and it has to be integrated with local base information [33]. Better prediction also means better prevention by developing more appropriate treatments tailored to the psychopathological dimensions associated with violence (e.g., impulsivity, hostility). If community psychiatry can prevent the violence associated with mental disorders, the full integration of patients and their families will be much easier.

## Supporting information

S1 Table Characteristics and activity data of 4 participating departments of mental health. (DOCX)

S2 Table BPRS-E exploratory factor analysis. (DOCX)

S3 Table Longitudinal evaluation of MOAS total score and subscales through generalized estimating equation (GEE) with interaction effect between time (from 1 to 24) and group. (DOCX)

S4 Table Generalized linear models without interaction effect between variables and group: predictors of any aggressive behavior (regardless of the two groups). (DOCX)

S1 Fig Longitudinal evaluation of MOAS Total score for males and females during the 1-year follow-up. Trend estimated through Smoothing Spline functions with corresponding 95% confidence bands.
(TIF)

S2 Fig MOAS Total score in the two months prior to acute hospital admission and after discharge. Longitudinal evaluation of MOAS Total score (n = 23 outpatients).
(TIF)

S1 File Dataset. (XLSX)
