# Supplementary material for: Violence risk and mental disorders (VIORMED-2): A prospective multicenter study in Italy
Source: PLoS One. 2019 Apr 16;14(4):e0214924. doi: 10.1371/journal.pone.0214924 (PMC6467378; doi:10.1371/journal.pone.0214924)
Supplement: S1 Table — (DOCX) [file pone.0214924.s001.docx]

**S1 Table.**

**Characteristics and activity data of 4 participating Departments of Mental Health**

| **DMH** | **CATCHMENT AREA (POULATION)** | **NUMBER OF COMMUNITY MENTAL HEALTH CENTRES** | **OVERALL NUMBER OF PATIENTS IN TREATMENT** |
| --- | --- | --- | --- |
| Brescia | 363,490 | 4 | 4,467 |
| Monza | 323,020 | 4 | 4,334 |
| Legnano | 398,954 | 4 | 4,435 |
| Garbagnate | 320,136 | 4 | 3,588 |
