# Supplementary material for: Violence risk and mental disorders (VIORMED-2): A prospective multicenter study in Italy
Source: PLoS One. 2019 Apr 16;14(4):e0214924. doi: 10.1371/journal.pone.0214924 (PMC6467378; doi:10.1371/journal.pone.0214924)
Supplement: S2 Table — (DOCX) [file pone.0214924.s002.docx]

| **Items** | **Affect-Anxiety** | **Activation** | **Negative Symptoms** | **Psychotic Symptoms** |
| --- | --- | --- | --- | --- |
| 1. Somatic concerns | **0.89** | -0.09 | 0.43 | 0.07 |
| 2. Anxiety | **1.02** | 0.25 | 0.14 | -0.03 |
| 3. Depression | **0.79** | -0.10 | 0.04 | -0.02 |
| 4. Suicidality | **0.23** | 0.12 | 0.00 | 0.02 |
| 5. Guilt | **0.47** | -0.16 | -0.38 | 0.14 |
| 6. Hostility | 0.28 | **0.90** | 0.01 | -0.06 |
| 7. Elevated mood | 0.09 | **0.40** | -0.11 | 0.09 |
| 8. Grandiosity | 0.06 | **0.59** | 0.17 | 0.09 |
| 9. Suspiciousness | 0.13 | **0.85** | 0.51 | 0.07 |
| 10. Hallucinations | 0.08 | 0.11 | -0.01 | **0.32** |
| 11. Unusual thought content | 0.13 | 0.16 | 0.30 | **0.74** |
| 12. Bizarre behaviour | 0.02 | 0.40 | 0.26 | **0.67** |
| 13. Self-neglect | 0.3 | 0.14 | 0.02 | **0.76** |
| 14. Disorientation | 0.04 | -0.01 | -0.01 | **0.15** |
| 15. Conceptual disorganization | 0.02 | -0.03 | 0.17 | **0.59** |
| 16. Blunted affect | 0.22 | -0.17 | **0.93** | 0.37 |
| 17. Emotional withdrawal | 0.20 | 0.00 | **1.09** | 0.20 |
| 18. Motor retardation | 0.33 | -0.15 | **0.48** | 0.27 |
| 19. Tension | **0.67** | 0.39 | 0.25 | -0.01 |
| 20. Uncooperativeness | 0.17 | **0.56** | -0.08 | 0.33 |
| 21. Excitement | 0.06 | **0.41** | -0.14 | 0.13 |
| 22. Distractibility | 0.11 | 0.18 | -0.06 | **0.63** |
| 23. Motor hyperactivity | 0.06 | **0.35** | -0.18 | 0.16 |
| 24. Mannerisms and posturing | 0.04 | 0.08 | 0.11 | **0.37** |
| BPRS-E=Brief Psychiatric Rating Scale - Expanded | | | | |

**S2 Table**

**BPRS-E exploratory factor analysis**
