# Supplementary material for: Violence risk and mental disorders (VIORMED-2): A prospective multicenter study in Italy
Source: PLoS One. 2019 Apr 16;14(4):e0214924. doi: 10.1371/journal.pone.0214924 (PMC6467378; doi:10.1371/journal.pone.0214924)
Supplement: S3 Table — (DOCX) [file pone.0214924.s003.docx]

**S3 Table**

**Longitudinal evaluation of MOAS Total Score and subscales through Generalized Estimating Equation (GEE) with interaction effect between time (from 1 to 24) and group**

|  | **MOAS**  **Total Score** | **MOAS**  **Verbal Aggression** | **MOAS**  **Aggression against objects** | **MOAS**  **Self- aggression** | **MOAS**  **Physical aggression** |
| --- | --- | --- | --- | --- | --- |
|  | *p value* | *p value* | *p value* | *p value* | *p value* |
| **Time** | <0.001 | <0.001 | <0.001 | <0.001 | <0.001 |
| **Group** | <0.001 | <0.001 | 0.010 | 0.057 | 0.013 |
| **Time x Group** | 0.055 | 0.191 | 0.002 | <0.001 | 0.006 |

MOAS=Modified Overt Aggression Scale
