# Supplementary material for: Violence risk and mental disorders (VIORMED-2): A prospective multicenter study in Italy
Source: PLoS One. 2019 Apr 16;14(4):e0214924. doi: 10.1371/journal.pone.0214924 (PMC6467378; doi:10.1371/journal.pone.0214924)
Supplement: S4 Table — (DOCX) [file pone.0214924.s004.docx]

**S4 Table**

**Generalized linear models without interaction effect between variables and group:**

**predictors of any aggressive behavior (regardless of the two groups)**

|  | β | *p value* | AIC |
| --- | --- | --- | --- |
| **Total MOAS**  BDHI Suspicion  BGLHA Total Score  STAXI Anger Expression Index  STAXI Trait Anger  STAXI Angry Temperament  STAXI Anger Expression-out  STAXI Anger Expression-in  STAXI Angry Reaction  Capability of collaboration in the last year *(Yes vs No)*  Economic independence *(Yes vs No)*  SLOF Activities  Use of substances in the last 12 months *(No vs Yes)*  Age of first contact with DMHs | 1.14  1.05  1.03  1.02  1.01  1.02  1.01  1.01  0.43  0.73  0.97  0.31  0.96 | 0.030  0.002  <0.001  <0.001  0.019  <0.001  0.032  0.006  0.039  0.021  0.021  0.001  0.001 | 1156.1  1208.9  1455.8  1507.0  1515.9  1529.1  1529.5  1533.9  1559.1  1564.3  1583.5  1586.7  1594.8 |
| **MOAS verbal aggression**  BDHI Suspicion  STAXI Trait Anger  STAXI Angry Temperament  STAXI State Anger  STAXI Feeling Angry  STAXI Feel like expressing anger verbally  STAXI Anger Expression-in  STAXI Anger Expression-out  STAXI Angry reaction  STAXI Feel like expressing anger physically  BPRS-E Affect Anxiety  Capability of collaboration in the last year *(Yes vs No)*  Use of substances in the last 12 months *(No vs Yes)*  Age of first contact with DMHs | 1.11  1.02  1.01  1.01  1.01  1.01  1.01  1.02  1.01  1.01  1.04  0.31  0.38  0.97 | 0.038  <0.001  0.005  0.004  0.008  0.006  0.011  <0.001  0.002  0.011  0.032  0.032  0.007  0.029 | 991.5  1260.5  1270.1  1270.2  1277.7  1279.0  1282.9  1284.2  1285.3  1286.6  1306.6  1310.2  1335.6  1343.5 |
| **MOAS aggression against objects**  STAXI Anger Expression Index  STAXI Trait Anger  Social Support in the last year *(Present vs Absent)*  Age of first contact with DMHs | 1.03  1.02  9.89  0.93 | 0.014  0.038  0.036  0.008 | 665.9  676.0  714.6  722.5 |
| **MOAS self-aggression**  BGLHA Total Score  BDHI Irritability  STAXI Anger Expression Index  SLOF Social acceptability/adjustment  SLOF Self-care  Sex *(Male vs Female)* | 1.15  1.92  1.06  0.79  1.35  0.15 | <0.001  0.008  0.003  0.019  0.023  0.011 | 239.7  245.6  320.9  336.8  362.7  363.4 |
| **MOAS physical aggression**  BGLHA Total Score | 1.10 | 0.018 | 443.7 |

BDHI=Buss-Durkee Hostility Inventory; BGLHA=Brown-Goodwin Lifetime History of Aggression; BPRS-E=Brief Psychiatric Rating Scale; MOAS=Modified Overt Aggression Scale; SLOF=Specific Levels Of Functioning; STAXI-2=Scale State-Trait Anger Expression Inventory 2.

β: estimate of the variable effect; *p value*: significance of the principal effect term; AIC: *Akaike Information Criterion* of the GLM.
