# Supplementary figures and images for: Violence risk and mental disorders (VIORMED-2): A prospective multicenter study in Italy
Source: PLoS One. 2019 Apr 16;14(4):e0214924. doi: 10.1371/journal.pone.0214924 (PMC6467378; doi:10.1371/journal.pone.0214924)

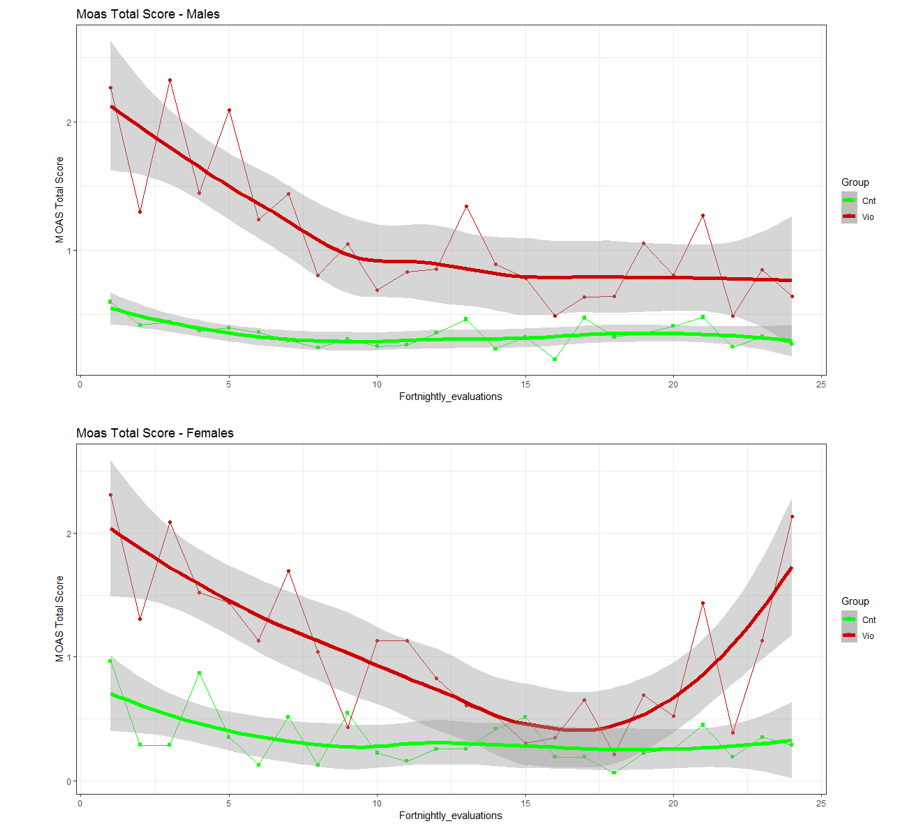

Supplement: S1 Fig — Trend estimated through Smoothing Spline functions with corresponding 95% confidence bands. (TIF) [file pone.0214924.s005.tif]

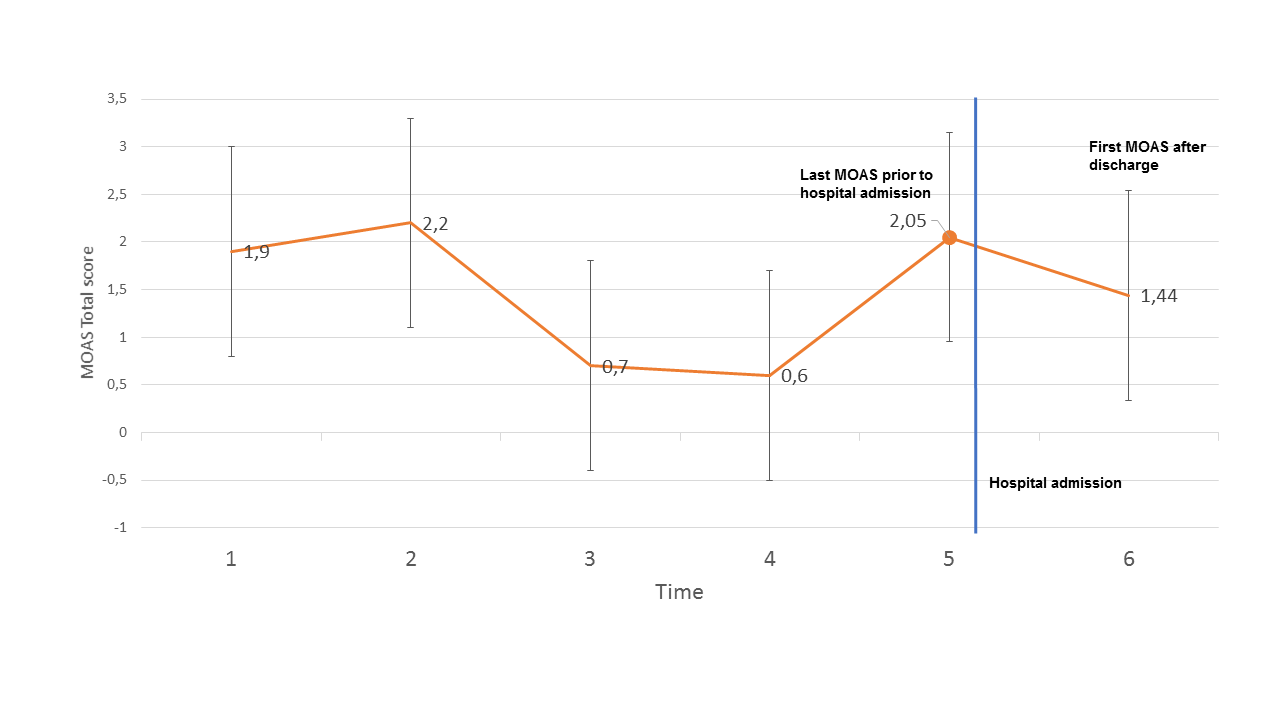

Supplement: S2 Fig — Longitudinal evaluation of MOAS Total score (n = 23 outpatients). (TIF) [file pone.0214924.s006.tif]
